# Recurrence of benign paroxysmal positional vertigo: risk factors and outcomes

**DOI:** 10.1590/1806-9282.20260695

**Published:** 2026-09-07

**Authors:** Ayhan Kars, Fatma Atalay, Sezai Sacid Anbar, Kübra Topal, Serkan Altıparmak

**Affiliations:** 1 Erzincan Binali Yıldırım University, Faculty of Medicine, Department of Otorhinolaryngology – Erzincan, Turkey.; 2 Kastamonu University, Faculty of Medicine, Department of Otorhinolaryngology – Kastamonu, Turkey.; 3 Kastamonu Training and Research Hospital, Department of Audiology – Kastamonu, Turkey.; 4 Private Otorhinolaryngology Clinic – Kastamonu, Turkey.

**Keywords:** Benign paroxysmal positional vertigo, Comorbidity, Hyperlipidemia, Recurrence, Risk factors

## Abstract

**OBJECTIVE:**

Benign paroxysmal positional vertigo is the most common peripheral vestibular disorder. It can recur despite successful maneuvers. While various risk factors for recurrence have been investigated, these remain controversial. The aim of this study was to identify potential risk factors for benign paroxysmal positional vertigo recurrence and contribute to the development of preventive treatment strategies.

**METHODS:**

The study involved 301 patients who presented to our clinic with dizziness and were diagnosed with benign paroxysmal positional vertigo between December 2019 and May 2022. Two groups, with and without benign paroxysmal positional vertigo recurrence, were compared in terms of age, gender, hypertension, diabetes mellitus, hyperlipidemia, cardiovascular disease, asthma, hypothyroidism, magnesium deficiency, vitamin D deficiency, vitamin B12 deficiency, and iron deficiency to investigate the effects of these factors on recurrence.

**RESULTS:**

Recurrence was observed in 38 (12.6%) of the 301 patients. No age or gender difference was determined between the groups. Univariate analysis showed that hypertension (55.3 vs. 29.3%; p=0.003), vitamin B12 deficiency (21.1 vs. 6.5%; p=0.007), hyperlipidemia (26.3 vs. 5.7%; p<0.001), and asthma (15.8 vs. 5.7%; p=0.035) were more common in patients with recurrence. Multivariate logistic regression analysis identified only hyperlipidemia as a risk factor, raising the risk of recurrence approximately 3.7-fold (OR 3.71; 95%CI 1.36–10.13; p=0.011).

**CONCLUSION:**

Consideration of underlying comorbidities and systemic factors, effective management of existing comorbidities, and individualized treatment strategies are crucial in order to reduce benign paroxysmal positional vertigo recurrence rates and improve patients’ quality of life.

## INTRODUCTION

Benign paroxysmal positional vertigo (BPPV) is the most common cause of peripheral vestibular vertigo. The condition is characterized by short-term, usually self-limiting vertigo and nystagmus that occur with positional movements of the head against gravity^1^. Two fundamental mechanisms have been identified to explain the pathophysiology of BPPV: the presence of free otoconia migrating from the utricular macula to the semicircular canals is known as canalolithiasis, and that of otoconia adhering to the cupula as cupulolithiasis^2^. BPPV is divided into two categories based on etiology: secondary BPPV has definite or probable causes such as secondary Ménière’s disease, sudden hearing loss, vestibular neuritis, otitis media, and head trauma, while primary or idiopathic BPPV involves an uncertain etiology^3^.

Although the exact prevalence of BPPV in the general population is unknown, the reported annual prevalence is 1.6%, the annual incidence is 0.6%, and the lifetime prevalence is 2.4%^4^. The prevalence of BPPV increases with age, and the incidence increases with acute or chronic illnesses^5^. The prevalence is reported to be as high as 10% in individuals over 75, and it is 2–3 times more common in women than in men^6^.

Canalith repositioning maneuvers are currently the most widely employed and effective method for treating BPPV. The average success rate of these maneuvers is between 70 and 90%^7^. However, despite successful treatment, approximately 50% of patients experience recurrence^8^. Predicting patients at risk for recurrence and identifying associated risk factors is therefore of considerable clinical importance. Although numerous demographic, metabolic, and clinical factors have been associated with recurrent BPPV, reappearance is considered multifactorial, and no validated clinical prediction model capable of reliably identifying patients at high risk of recurrence has yet emerged^9,10^.

The aim of this study was to identify comorbid factors that exacerbate the risk of recurrence in patients with BPPV and to contribute to the development of treatments and clinical strategies needed to prevent recurrence.

## METHODS

Approval for this retrospective study was obtained from the Kastamonu University Clinical Research Ethics Committee (Approval No. 2022/67, Date: 06.07.2022). All procedures were conducted in accordance with the institutional ethical standards and the 1964 Declaration of Helsinki. Due to the retrospective study design and the use of anonymized data, individual informed consent was not required.

Patients aged 18 and over who presented to the Kastamonu University Ear, Nose, and Throat (ENT) Clinic between December 2019 and May 2022 with complaints of dizziness and who were diagnosed with BPPV through canalith repositioning maneuvers were included in the study. All patients underwent detailed ENT examinations, and those with acute or chronic otitis media were excluded from the study. Patients with peripheral or central vertigo other than BPPV, with intracranial masses, with previous histories of ear surgery, or using chemotherapy or ototoxic drugs, pregnant women, and patients whose detailed medical records were not available were also excluded. Seventeen patients were excluded because information regarding comorbidities and laboratory parameters required for the analysis was not available in the medical records.

The demographic and clinical characteristics of the patients enrolled were recorded, including age, gender, date of first presentation, the affected side and semicircular canal, and the dates of attacks occurring during follow-up. Comorbid conditions such as hypertension, diabetes mellitus, hyperlipidemia, cardiovascular disease, asthma, hypothyroidism, and magnesium, vitamin D, vitamin B12, or iron deficiency were also recorded. Information regarding comorbidities and laboratory-based variables was obtained from medical records available prior to or at the time of diagnosis of BPPV. The average follow-up period was 2 years.

Diagnostic and treatment protocols were applied in accordance with the clinical practice guidelines for BPPV established by the American Academy of Otolaryngology-Head and Neck Surgery. Dix-Hallpike maneuvers were used for the posterior canal, roll maneuvers for the lateral canal, and supine head-hanging maneuvers for the anterior canal. Therapeutic maneuvers included the Epley maneuver for the posterior canal, the Barbecue or Gufoni maneuver for the lateral canal, and the deep head hanging (Yacovino) maneuver for the anterior canal. All maneuvers were performed by an experienced vestibular technician.

If more than one semicircular canal was affected, appropriate maneuvers were applied depending the severity of vertigo, nystagmus, and the type of canal involved. Recurrence was defined as the reappearance of ipsilateral BPPV following a symptom-free period of at least one week following successful treatment and was confirmed by positional testing. No contralateral recurrences were observed during the study period. Recurrences were identified when patients re-presented to the ENT clinic with recurrent positional vertigo during follow-up and recurrence was confirmed by positional testing. Routine scheduled follow-up visits were not required for all patients.

### Statistical analysis

Statistical analyses of the data obtained in this study were performed using IBM Statistical Package for the Social Sciences Statistics version 26.0 software (IBM Corporation, New York, NY, USA). The distribution of continuous variables was assessed using visual methods (histograms) and analytical tests. Continuous variables were expressed as mean±standard deviation or median (minimum–maximum) according to their conformity with normal distribution, while categorical variables were expressed as numbers and percentages. Comparisons between groups with and without recurrence were conducted using the Mann-Whitney U test for continuous variables, and the chi-square test or Fisher’s Exact Test, as appropriate, for categorical variables. Univariate analyses were first performed to identify variables potentially associated with recurrence. Due to the limited number of recurrence events and the potential risk of model overfitting, the final multivariable logistic regression model was restricted to three clinically relevant predictors (hypertension, hyperlipidemia, and vitamin B12 deficiency). Regression results were reported as odds ratios (ORs) with 95%CIs. The discriminatory power of the regression model was evaluated using receiver operating characteristic (ROC) curve analysis, and the area under the curve (AUC) was calculated. Internal validation was performed using 1,000 bootstrap resamples. Model calibration was evaluated using a calibration plot (Supplementary Figure 1) and the Hosmer-Lemeshow goodness-of-fit test. Multicollinearity between variables was assessed using variance inflation factors (VIFs). A two-sided p<0.05 was regarded as significant.

### Declaration of generative artificial intelligence and artificial intelligence-assisted technologies in the manuscript preparation process

During the preparation and revision of this manuscript, the authors used ChatGPT-5 exclusively for language editing and the improvement of manuscript readability. The authors reviewed and edited the content as required and assume full responsibility for the content of the manuscript.

## RESULTS

Three hundred one patients were included in the study, 38 (12.6%) of whom experienced recurrence. The mean age of the two groups was 53.7±16.1 years, and 59.1% of the patients were women. The most commonly affected semicircular canal was the posterior canal (88.7%). The rate of bilateral canal involvement was 6%.

The mean time to recurrence from the first attack was 6.7±5.8 months, and the median time was 4.25 months (minimum-maximum: 0.5–20 months). These results indicate that the majority of recurrences occur within the first year. The first recurrence occurred within the first six months in 55.3% of cases, and within the first year in 84.2%. Of the 38 patients who developed recurrence, 68.4% (n=26) experienced a single attack, 28.9% (n=11) two attacks, and 2.6% (n=1) three or more attacks.

No statistically significant difference was observed between the recurrence and non-recurrence groups in terms of gender or channel distribution (p>0.05). Although mean age was higher among the patients with recurrence, the difference was not statistically significant (p=0.070) (Table 1).

**Table 1:** Demographic and canal characteristics of patients with and without recurrence of benign paroxysmal positional vertigo.

Variable	Total (n=301)	No recurrence (n=263)	Recurrence (n=38)	p-value
Age (years), mean±SD	53.7±16.1	53.1±16.2	57.7±14.6	0.070
Female, n (%)	178 (59.1)	156 (59.3)	22 (57.9)	0.862
Canal type, n (%)				
Posterior canal	267 (88.7)	233 (88.6)	34 (89.5)	0.832
Lateral canal	6 (2.0)	5 (1.9)	1 (2.6)	
Anterior canal	3 (1.0)	2 (0.8)	1 (2.6)	
Multi-canal involvement	6 (2.0)	5 (1.9)	1 (2.6)	
Bilateral posterior	18 (6.0)	16 (6.1)	2 (5.3)	

SD, standard deviation. p-value for canal distribution: chi-square test.

Univariate analysis revealed that hypertension (55.3 vs. 29.3%; p=0.003), vitamin B12 deficiency (21.1 vs. 6.5%; p=0.007), hyperlipidemia (26.3 vs. 5.7%; p<0.001), and asthma (15.8 vs. 5.7%; p=0.035) were significantly more common in patients with recurrence. Although diabetes mellitus was also observed at a higher rate in the recurrence group, the difference was not statistically significant (p=0.061). Age was higher in the recurrence group, but this was also not statistically significant (p=0.070). No significant difference was determined between the groups in terms of hypothyroidism, magnesium deficiency, vitamin D deficiency, iron deficiency, or cardiovascular disease (p>0.05) (Table 2).

**Table 2:** Univariate analysis of factors associated with recurrence of benign paroxysmal positional vertigo.

Variable	No recurrence (n=263)	Recurrence (n=38)	p-value
Age (years), mean±SD	53.1±16.2	57.7±14.6	0.070
Hypertension, n (%)	77 (29.3%)	21 (55.3%)	0.003
Diabetes mellitus, n (%)	27 (10.3%)	8 (21.1%)	0.061
Hypothyroidism, n (%)	25 (9.5%)	3 (7.9%)	1.000
Magnesium deficiency, n (%)	12 (4.6%)	4 (10.5%)	0.128
Vitamin D deficiency, n (%)	122 (46.4%)	19 (50.0%)	0.730
Vitamin B12 deficiency, n (%)	17 (6.5%)	8 (21.1%)	0.007
Iron deficiency, n (%)	3 (1.1%)	2 (5.3%)	0.121
Cardiovascular disease, n (%)	71 (27.0%)	15 (39.5%)	0.126
Hyperlipidemia, n (%)	15 (5.7%)	10 (26.3%)	<0.001
Asthma, n (%)	15 (5.7%)	6 (15.8%)	0.035

SD, standard deviation. Categorical variables were compared using the chi-square or Fisher’s exact test, as appropriate. Continuous variables were compared using the Mann-Whitney U test.

Multivariable logistic regression analysis identified hyperlipidemia as the only independent factor significantly associated with BPPV recurrence, exhibiting approximately 3.9-fold higher odds of recurrence compared with patients without hyperlipidemia (OR 3.85; 95%CI 1.48–10.01; p=0.006). Although patients with hypertension exhibited higher odds of recurrence, this association did not reach statistical significance (OR 2.04; 95%CI 0.96–4.34; p=0.065). Vitamin B12 deficiency was also not independently associated with recurrence (OR 2.19; 95%CI 0.79–6.06; p=0.130) (Table 3).

**Table 3:** Multivariable logistic regression analysis of factors associated with benign paroxysmal positional vertigo recurrence.

Variable	Odds ratio (OR)	95%CI	p-value
Hypertension	2.04	0.96–4.34	0.065
Hyperlipidemia	3.85	1.48–10.01	0.006
Vitamin B12 deficiency	2.19	0.79–6.06	0.130

CI: confidence interval; OR: odds ratio. Binary logistic regression model. The multivariable model was restricted to three predictors to reduce the risk of overfitting due to the limited number of recurrence events. Internal validation was performed using 1,000 bootstrap resamples. The apparent AUC was 0.671, and the optimism-corrected AUC was 0.661.

The performance of the logistic regression model was assessed using ROC curve analysis. The model demonstrated acceptable discriminatory power (apparent AUC=0.671; optimism-corrected AUC=0.661). Its fit to the data was assessed using the Hosmer-Lemeshow goodness-of-fit test, which demonstrated good calibration (p=0.423). The calibration analysis also showed good agreement between predicted and observed probabilities of recurrence (Supplementary Figure 1). Multicollinearity between variables was examined using VIF analysis, and all VIF values were below 2.

## DISCUSSION

Recurrence rates for BPPV are known to vary depending on follow-up duration, diagnostic criteria, and patient characteristics. In a prospective study of 548 patients, Kong et al. reported a recurrence rate of 22.1%, with approximately 70% of recurrences occurring within the first year^11^. In the present study, recurrence occurred in 12.6% of patients, and the majority of recurrences were also observed during the first year, consistent with previous reports.

Some studies have reported a significant effect of age and gender on BPPV recurrence, although others have reported contrary findings. Chen et al.^12^ examined a large number of clinical and demographic characteristics in their meta-analysis and reported that age and gender emerged as significant in some studies, but there was significant heterogeneity among the studies and the results were not consistent. Although mean age in the present study was higher in the recurrence group, the difference was not found to be statistically significant. No significant gender difference was also determined between the two groups.

Increasing evidence suggests that vascular, metabolic, and autoimmune comorbidities may affect otolith stability and contribute to BPPV recurrence^13^. The assessment of concomitant systemic diseases may therefore contribute to a more comprehensive clinical evaluation of affected patients. While hypertension, vitamin B12 deficiency, hyperlipidemia, and asthma were associated with recurrence at univariate analysis in this study, only hyperlipidemia remained significantly associated with recurrence at multivariate analysis. This finding is consistent with previous studies identifying hyperlipidemia as a potential risk factor for BPPV recurrence and therefore supports the growing body of evidence linking metabolic dysfunction to recurrent disease^13^. Hyperlipidemia may impair the microvascular circulation of the inner ear, resulting in ischemia and degeneration of the otolithic organs, thereby contributing to vestibular dysfunction and BPPV recurrence^14^. Recent evidence suggests that BPPV recurrence is multifactorial and may be affected by metabolic and vascular comorbidities, including hypertension, diabetes mellitus, and hyperlipidemia. These conditions are thought to contribute to recurrence through impaired inner ear microcirculation and alterations in otolith metabolism^15^. Although hyperlipidemia remained significantly associated with recurrence in the multivariable analysis in the present study, the confidence interval around the OR was relatively wide. This finding may reflect the limited number of recurrence events and patients with hyperlipidemia in the present cohort and should therefore be interpreted with caution. Larger prospective studies are now needed to further validate the magnitude of this association.

No significant association was observed between diabetes mellitus, hypothyroidism, cardiovascular disease, vitamin D deficiency, magnesium, or iron deficiencies and BPPV recurrence in the present research. While some publications have suggested that cardiovascular diseases and diabetes mellitus may affect microcirculation in the vestibular system and cause recurrence, other studies have reported that metabolic and vascular comorbidities alone cannot be considered strong predictors^11^. Although some studies have implicated vitamin D deficiency in both the etiology and recurrence of BPPV, Li et al.^16^ concluded that vitamin D deficiency may play a role in the development of BPPV, but not in recurrence. The lack of a significant association in the current study is consistent with these findings and may be attributable to differences in study populations, definitions of vitamin D deficiency, follow-up durations, and adjustment for potential confounding factors across studies. Recent evidence further supports the multifactorial nature of BPPV recurrence. A recent meta-analysis identified advanced age, diabetes mellitus, female gender, vitamin D deficiency, migraine, osteoporosis, head trauma, and Meniere’s disease as significant factors associated with recurrence^17^. These findings suggest that comorbid conditions alone are not a determining factor in BPPV recurrence and that recurrence may involve a multifactorial pathophysiology with different mechanisms. Metabolic and vascular factors should be considered in clinical practice, but should not be regarded as sole predictive factors for recurrence.

There are a number of limitations to this study. First, due to the retrospective study design, patient information was obtained from medical records, which may have resulted in incomplete or inaccurate documentation of some variables. In addition, follow-up intervals were not fully standardized, treatment adherence for comorbid conditions could not be assessed, and selection bias cannot be completely excluded. The relatively small number of patients experiencing recurrence may also have affected the generalizability of the results. Due to the limited number of recurrence events, post hoc power analysis indicated that the study was adequately powered to detect only moderate-to-large effect sizes, and smaller but potentially clinically relevant associations may therefore have remained undetected. Furthermore, several potentially relevant confounding factors, including dietary habits, history of head trauma, medication use, migraine, osteoporosis, body mass index, physical activity, and socioeconomic status, were not evaluated and may have affected the observed associations. In addition, information regarding lipid-lowering therapies, including statin use, was not consistently available and therefore could not be evaluated. The observed association between hyperlipidemia and BPPV recurrence should consequently be interpreted with caution, since the effects of hyperlipidemia itself could not be distinguished from the potential effects of its treatment. Furthermore, patients with missing comorbidity or laboratory data were excluded before the final study cohort was established. The analyses were thus performed using a complete-case approach, and multiple imputation was not applied. Finally, since factors such as obesity, physical activity, dietary habits, and other components of metabolic syndrome were not evaluated, residual confounding cannot be completely excluded.

## CONCLUSION

The recurrence of BPPV appears to be a multifactorial process that cannot be explained by any single factor, with metabolic and vascular events being particularly prominent. The fact that most recurrences occur within the first year means that these patients require close follow-up. Hyperlipidemia was significantly associated with recurrence at multivariate analysis in this study. Lipid profile evaluation and assessment of metabolic risk factors may be regarded as a component of the comprehensive clinical evaluation of patients with recurrent BPPV.

## Data Availability

The datasets generated and/or analyzed during the current study are available from the corresponding author upon reasonable request.
